# Supplementary material for: Green synthesis of graphite from CO2 without graphitization process of amorphous carbon
Source: Nat Commun. 2021 Jan 5;12:119. doi: 10.1038/s41467-020-20380-0 (PMC7785740; doi:10.1038/s41467-020-20380-0)
Supplement: Supplementary file 1 — Supplementary Information [file 41467_2020_20380_MOESM1_ESM.pdf]

Supplementary information

Green synthesis of graphite from CO_2 without graphitization process of amorphous carbon

Chu Liang[†1,2,3], Yun Chen[†1], Min Wu[1], Kai, Wang[1], Wenkui Zhang[1]*, Yongping Gan[1], Hui Huang[1], Jian Chen[4], Yang Xia[1], Jun Zhang[1], Shiyou Zheng[2]*, Hongge Pan[3]*

[1] College of Materials Science and Engineering, Zhejiang University of Technology, Hangzhou 310014, China. [2] School of Materials Science and Engineering, University of Shanghai for Science and Technology, Shanghai 200093, China. [3] School of Materials Science and Engineering & State Key Lab of Silicon Materials, Zhejiang University, Hangzhou 310007, China. [4] Institute of Science and Technology for New Energy, Xi'an Technological University, Xi'an, 710021, China.

[†] These authors contributed equally to this work. Correspondence and requests for materials should be addressed to W.Z. (email: mseche m@zjut.edu.cn), S.Z. (email: syzheng@usst.edu.cn) or H.P. (email: hgpan@zju.edu.cn)

1. Supplementary figures

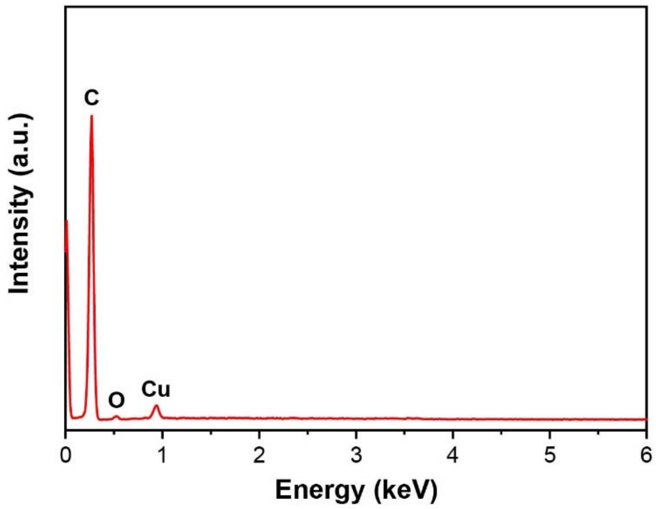

Supplementary figure 1 EDS spectrum of the as-obtained black powders.

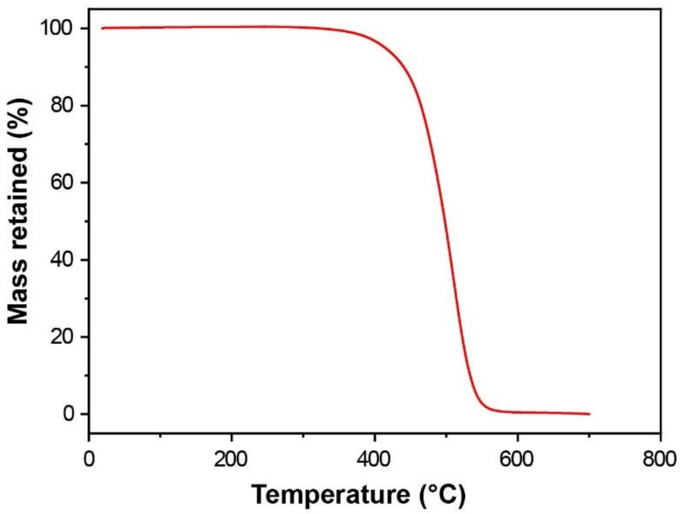

Supplementary figure 2 TG curve of as-obtained black powders.

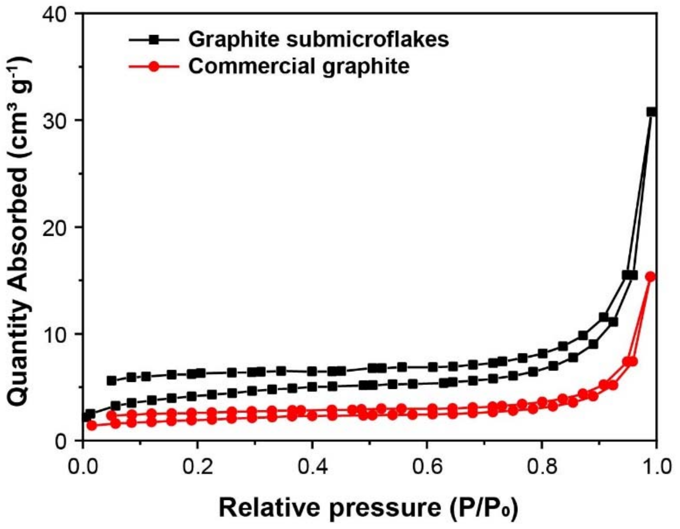

Supplementary figure 3 The specific surface area of graphite submicroflakes and commercial graphite.

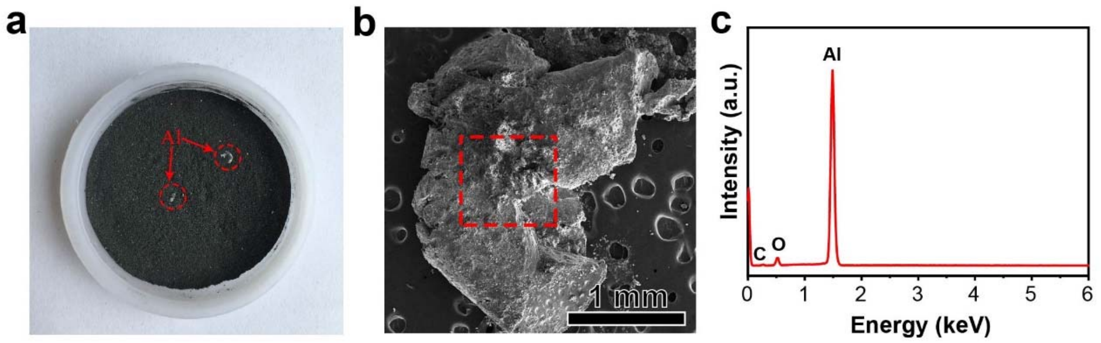

Supplementary figure 4 **a** Picture of solid products. **b** SEM image and **c** EDS spectrum of particle aggregation of Al.

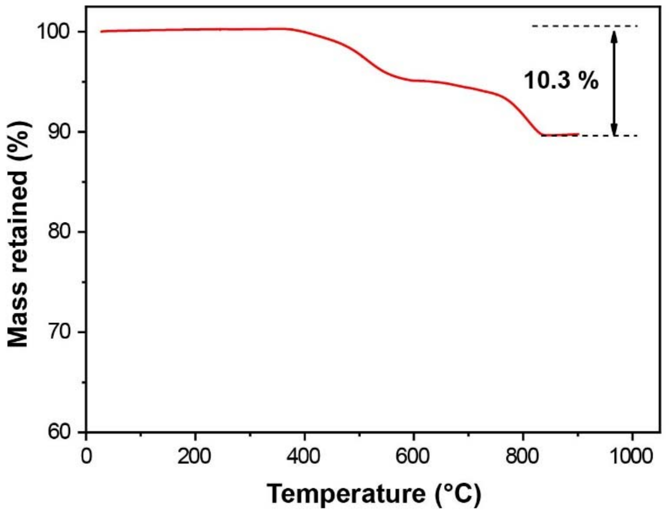

Supplementary figure 5 TG curves of the solid products of CO_2 reacting with $LiAlH_4$ heated under air.

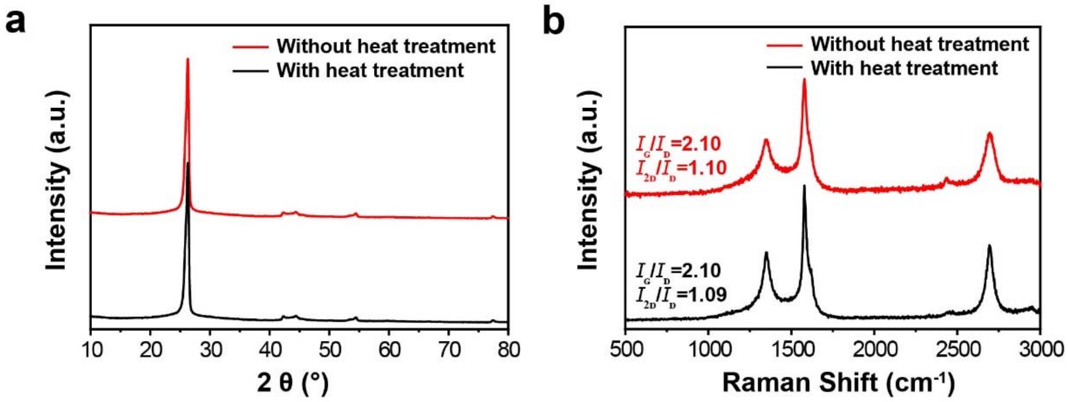

Supplementary figure 6 XRD patterns (**a**) and Raman spectra (**b**) of the carbon synthesized by reacting CO_2 with $LiAlH_4$ with and without heat treatment at 880 C.

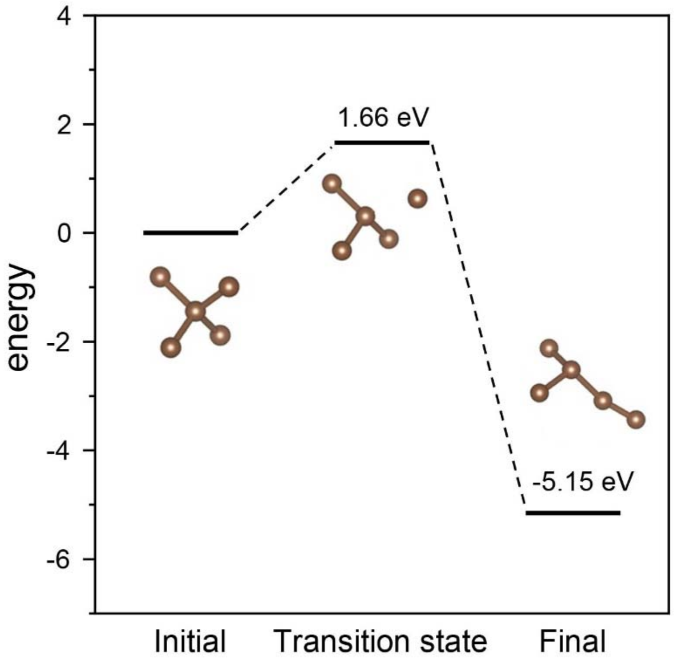

Supplementary figure 7 The energy profile for a sp[3] hybridized C cluster transforming to a planar sp[2] hybridized C cluster

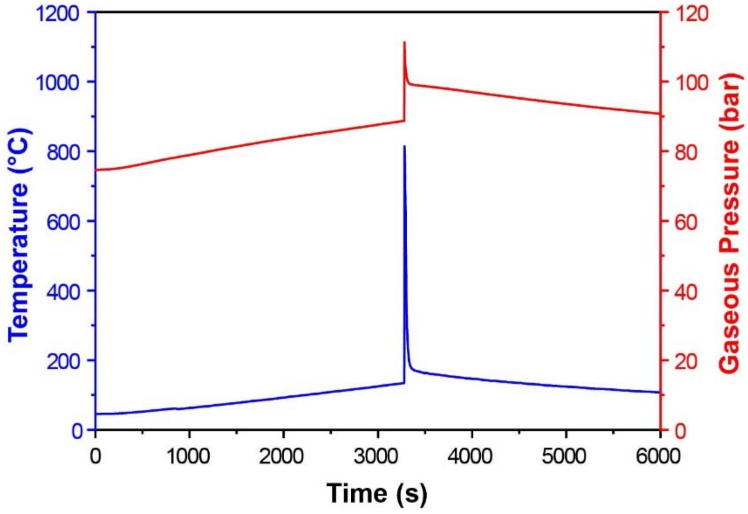

Supplementary figure 8 Time dependence of temperature and gas pressure in the reactor during 35 bar CO_2 reacting with $LiAlH_4$ under 75 bar gaseous back pressure. (35 bar CO_2+40 bar Ar)

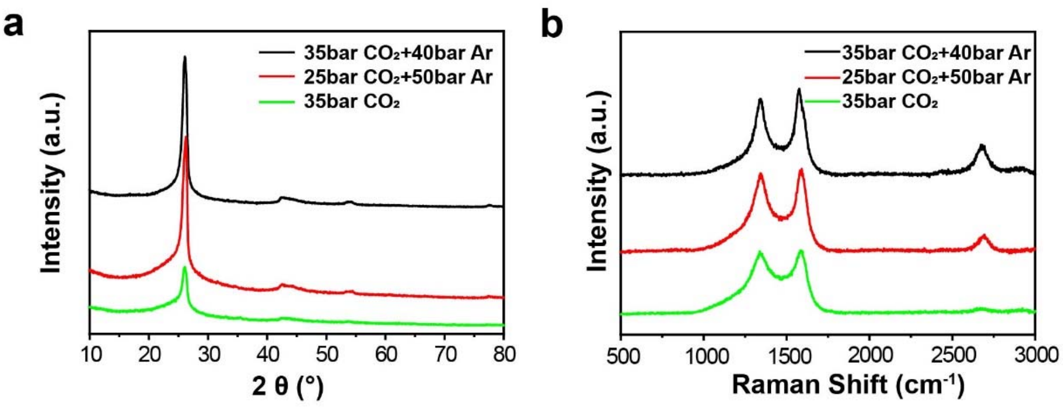

Supplementary figure 9 XRD patterns (**a**) and Raman spectra (**b**) of the carbon synthesized by reacting 25 bar CO_2 with $LiAlH_4$ under 75 bar gaseous back pressure.

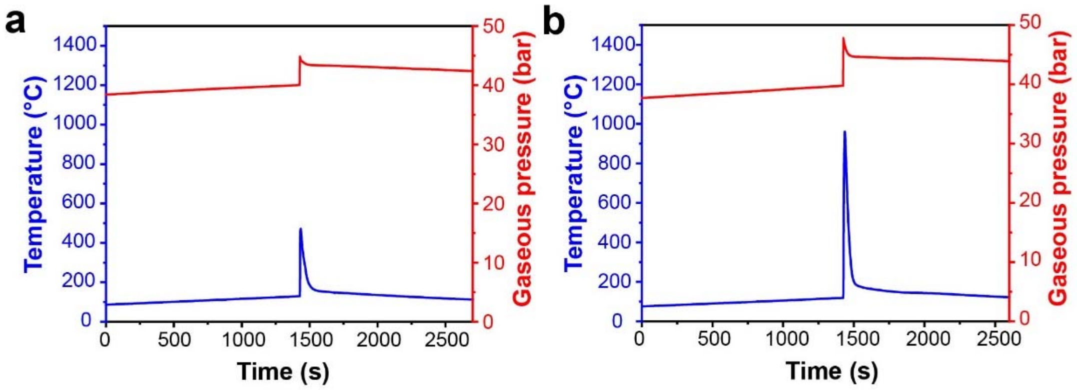

Supplementary figure 10 Time dependence of temperature and gas pressure in the reactor during 35 bar CO_2 reacting with $LiAlH_4$. **a** 0.30 g $LiAlH_4$, **b** 0.50 g $LiAlH_4$.

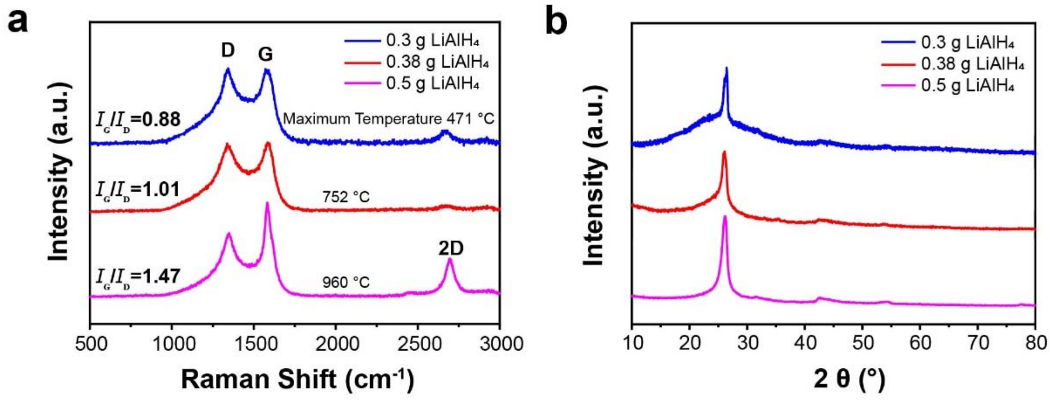

Supplementary figure 11 Raman spectra (**a**) and XRD patterns (**b**) of the carbon synthesized by reacting 35 bar CO_2 with 0.3, 0.38 and 0.5 g $LiAlH_4$.

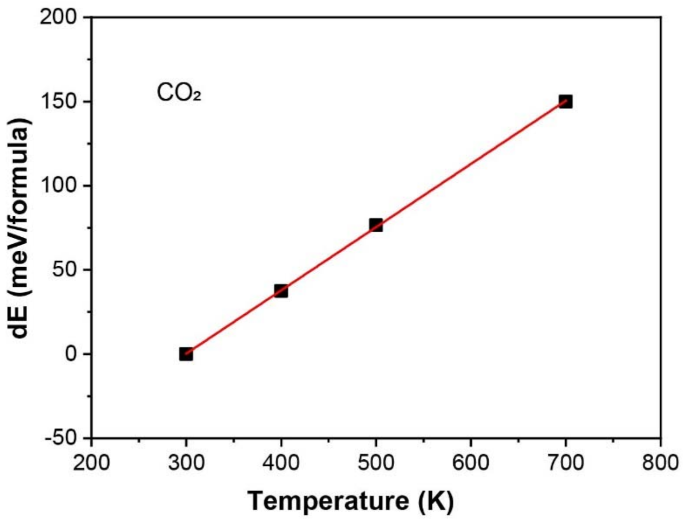

Supplementary figure 12 Effect of temperatures on the energy of CO_2 determined by first-principles molecular dynamics (FPMD) calculations.

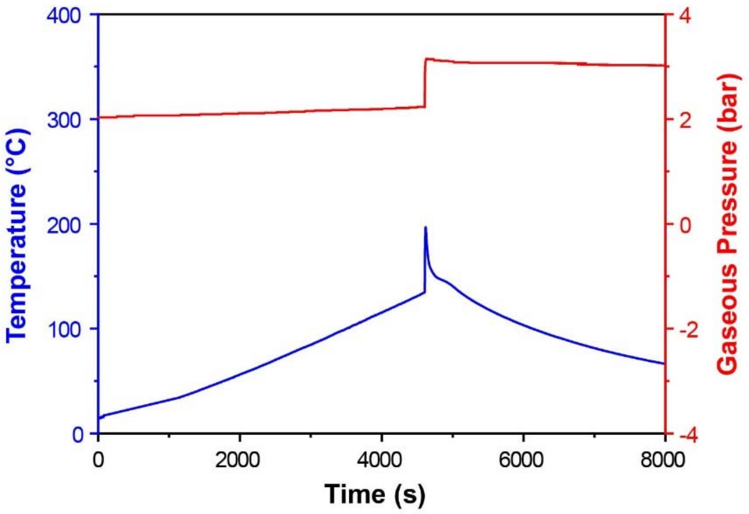

Supplementary figure 13 Time dependence of temperature and gas pressure in the reactor during 2 bar CO_2 reacting with $LiAlH_4$.

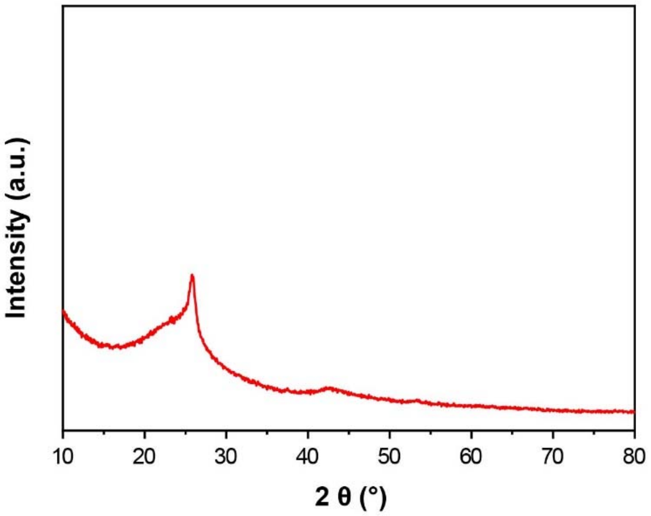

Supplementary figure 14 XRD pattern of the carbon synthesized by reacting 2 bar CO_2 with $LiAlH_4$.

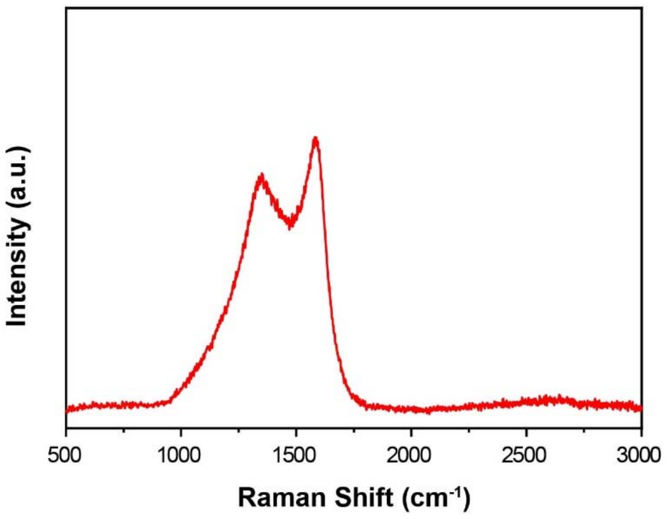

Supplementary figure 15 Raman spectrum of the carbon synthesized by reacting 2 bar CO_2 with $LiAlH_4$.

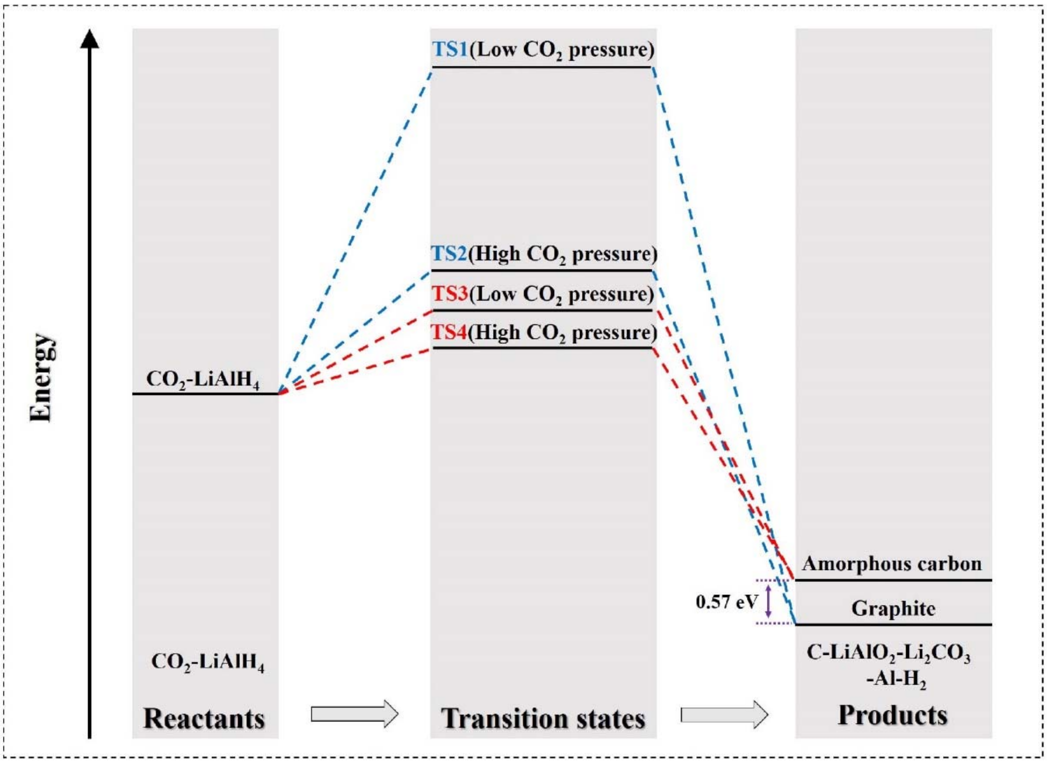

Supplementary figure 16 Schematic illustration of thermodynamics and kinetics of the reaction for synthesizing graphite and amorphous carbon from CO_2 (Supplementary note 1).

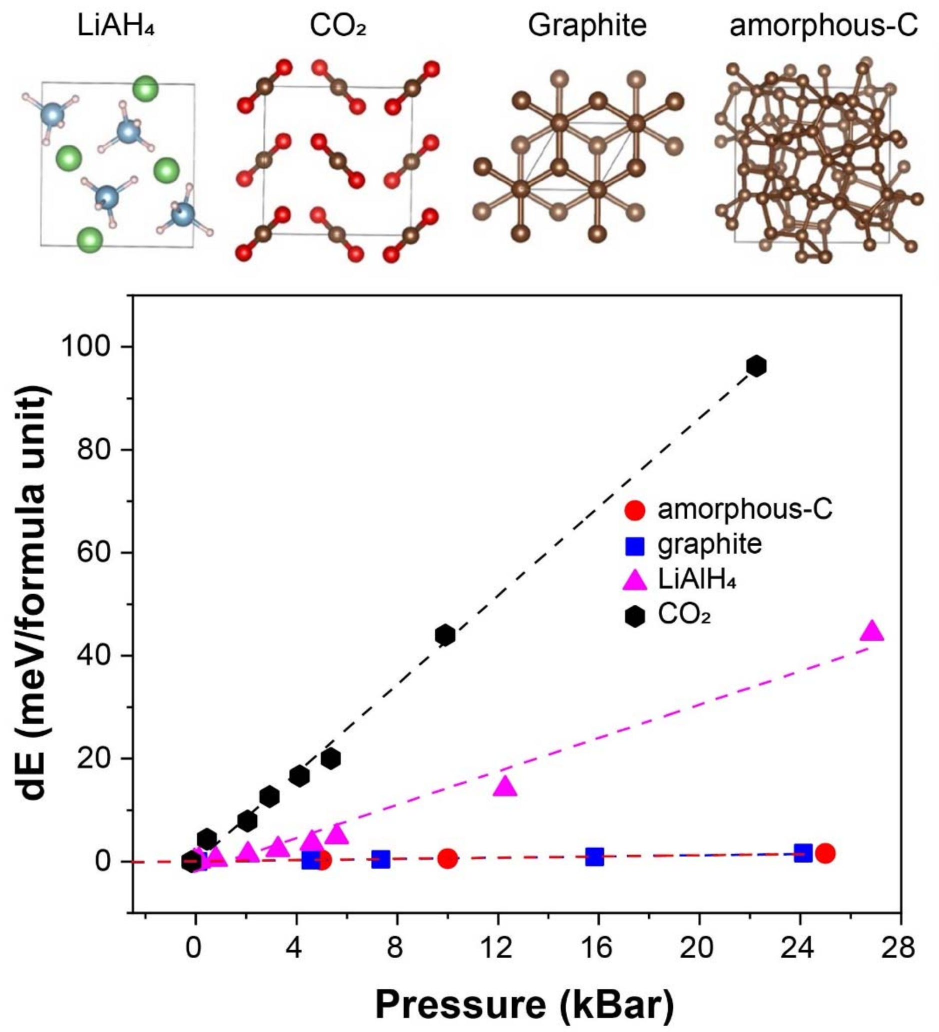

Supplementary figure 17 The structural models of $LiAlH_4$, CO_2, Graphite and amorphous-C, and their pressure dependent energy differences. The green, grey, white, brown and red balls in the structural models represent the Li, Al, H, C and O atoms, respectively.

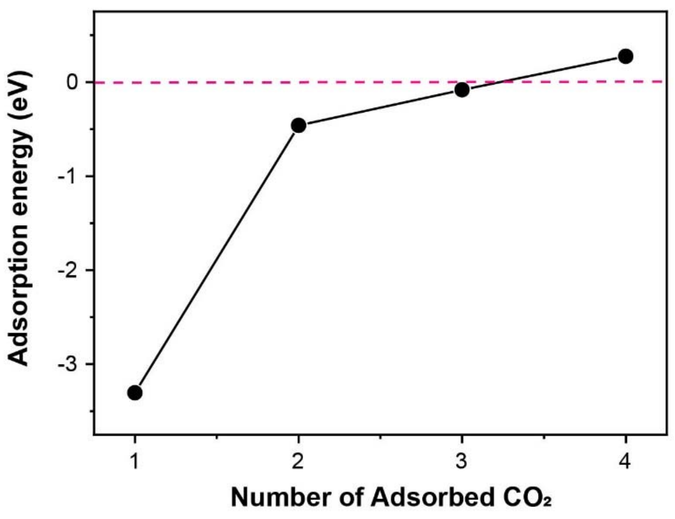

Supplementary figure 18 Adsorption energies of sequentially adsorbed CO_2 molecules on the (100) surface $LiAlH_4$.

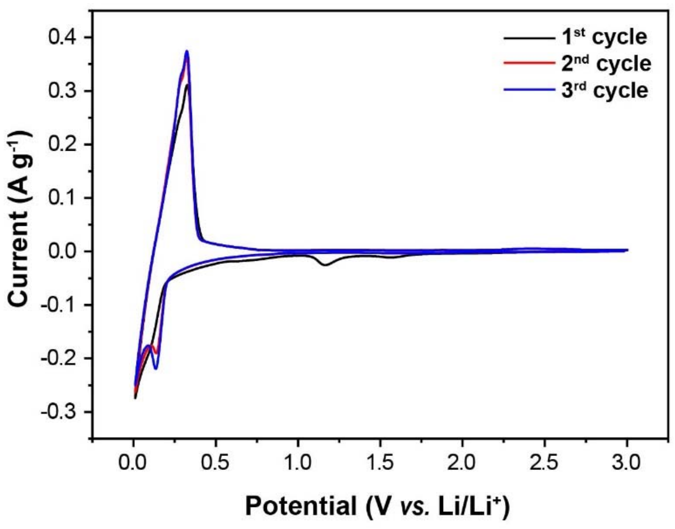

Supplementary figure 19 CV curves of commercial graphite.

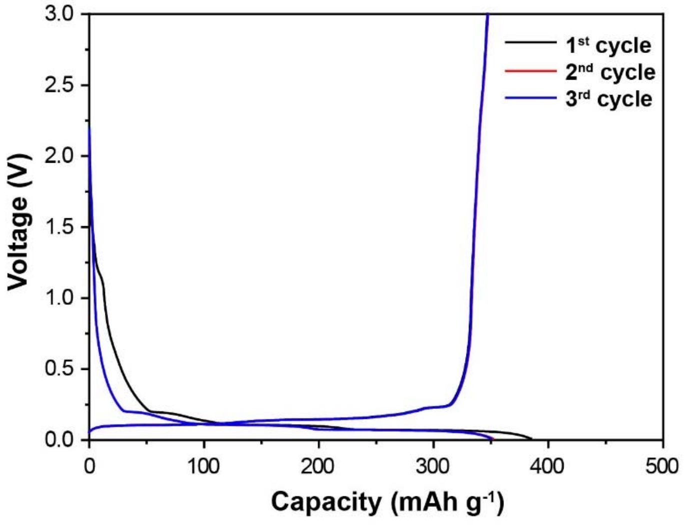

Supplementary figure 20 Discharge-charge curves of commercial graphite.

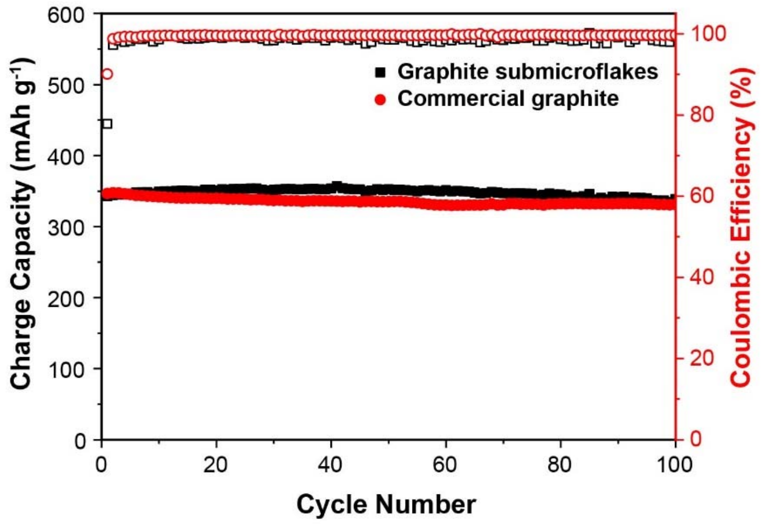

Supplementary figure 21 Cycling performance and Coulombic efficiency of graphite submicroflakes and commercial graphite from 1st to 100th cycles at 0.1 A g^{-1}.

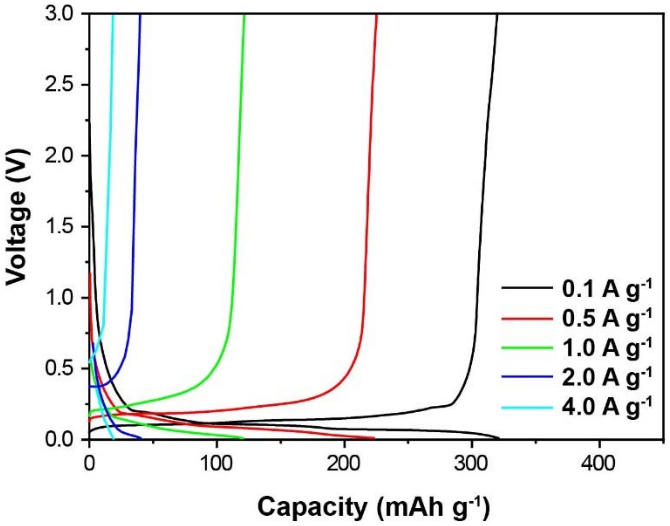

Supplementary figure 22 Discharge-charge curves of commercial graphite from 0.1 to 4.0 A g^{-1}.

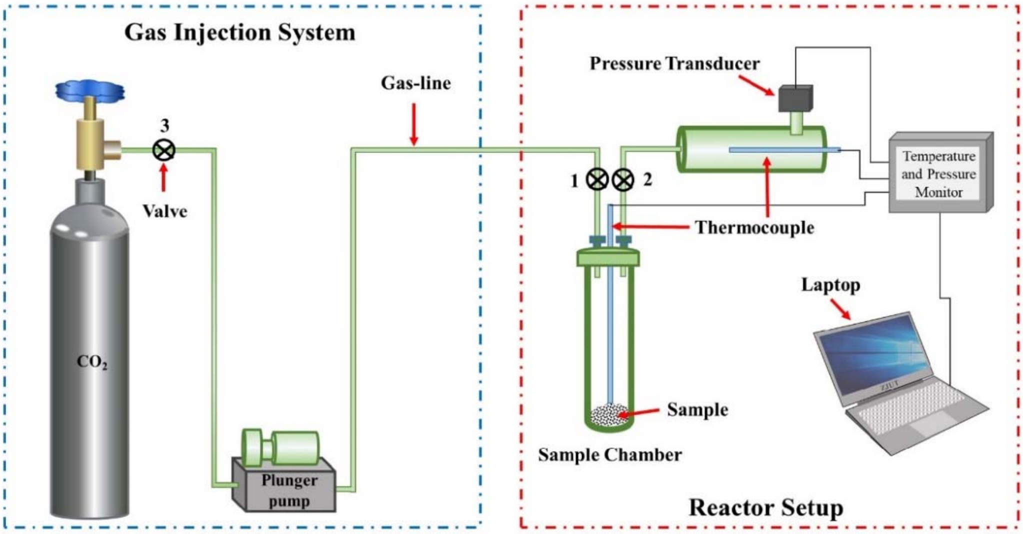

Supplementary figure 23 Schematic diagram of home-made reactor setup and CO_2 injection system.

2. Supplementary tables

Supplementary table 1 Content of elements in the as-obtained black powders

Elements	Content/wt.%
C	97.27
O	2.73

The content of elements was determined by EDS.

Supplementary table 2 Content of metal ions in as-synthesized graphite submicroflakes and commercial graphite

	Li/wt.%	Al//wt.%	K/wt.%	Other metals/wt.%
Graphite Submicroflakes	0.0038	0.0034	0.0030	0.0018
Commercial Graphite	0	0.00007	0.0026	0.0009

The content of metal ions was determined by ICP-MS.

Supplementary table 3 Content of elements in particle aggregation of Al.

Elements	Content/wt.%
Al	78.31
C	8.84
O	12.85

The content of elements was determined by EDS.

3. Supplementary notes

Supplementary note 1 The schematic illustration of supplementary figure 16 was plotted based on the experimental results combined with DFT calculation.

The energy of products (graphite/amorphous carbon, $LiAlO_2$, Li_2CO_3, Al and H_2) is lower than that of reactants (CO_2-$LiAlH_4$ system) since the exothermic nature was demonstrated for the synthesis reaction of graphite/amorphous carbon. Our DFT calculation shows that the energy difference between amorphous carbon and graphite is –0.57 eV/formula, implying that graphite is more thermodynamically stable than amorphous carbon. The energy difference between transition state and reactants was estimated by the following experimental results. The initial CO_2 pressures are 2 bar to 35 bar, 75 bar (liquid CO_2) at room temperature for synthesizing carbon. During heating, the corresponding CO_2 pressure at the initial reaction temperature was determined to be 2.2 bar at 142 °C (Figure S13), 42 bar at 128 °C (Figure 3d), and 132 bar at 126 °C (Figure 1b), respectively. The amorphous carbon was formed at the initial stage of synthesis reaction under low and high pressures. The above results indicate the kinetic barrier of the synthesis reaction of amorphous carbon is gradually reduced with the CO_2 pressure. Moreover, the graphite with good crystalline was only produced at the second stage of synthesis reaction under high pressure, in which the maximum pressure is 168 bar at maximum temperature of 876 °C (Figure 1b), the kinetic barrier for synthesizing graphite can be remarkably reduced under high gas pressure. Compared with amorphous carbon, the reduction in kinetic barrier for synthesizing graphite is much greater than that of amorphous carbon whereas the

kinetic barrier for synthesizing graphite is still higher than that for synthesizing amorphous carbon.
